# The coding/non-coding overlapping architecture of the gene encoding the Drosophila pseudouridine synthase

**DOI:** 10.1186/1471-2199-8-15

**Published:** 2007-02-28

**Authors:** Sara Riccardo, Giuseppe Tortoriello, Ennio Giordano, Mimmo Turano, Maria Furia

**Affiliations:** 1 Department of Structural and Functional Biology, University of Naples "Federico II", Complesso Universitario Monte Santangelo via Cinthia, 80126 Napoli, Italy

## Abstract

**Background:**

In eukaryotic cells, each molecule of H/ACA small nucleolar RNA (snoRNA) assembles with four evolutionarily conserved core proteins to compose a specific ribonucleoprotein particle. One of the four core components has pseudouridine synthase activity and catalyzes the conversion of a selected uridine to pseudouridine. Members of the pseudouridine synthase family are highly conserved. In addition to catalyzing pseudouridylation of target RNAs, they carry out a variety of essential functions related to ribosome biogenesis and, in mammals, to telomere maintenance. To investigate further the molecular mechanisms underlying the expression of pseudouridine synthase genes, we analyzed the transcriptional activity of the Drosophila member of this family in great detail.

**Results:**

The Drosophila gene for pseudouridine synthase, *minifly/Nop60b *(*mfl*), encodes two novel mRNAs ending at a downstream poly(A) site. One species is characterized only by an extended 3'-untranslated region (3'UTR), while a minor mRNA encodes a variant protein that represents the first example of an alternative subform described for any member of the family to date. The rare spliced variant is detected mainly in females and is predicted to have distinct functional properties. We also report that a cluster comprising four isoforms of a C/D box snoRNA and two highly related copies of a small ncRNA gene of unknown function is intron-encoded at the gene-variable 3'UTRs. Because this arrangement, the alternative 3' ends allow *mfl *not only to produce two distinct protein subforms, but also to release different ncRNAs. Intriguingly, accumulation of all these intron-encoded RNAs was found to be sex-biased and quantitatively modulated throughout development and, within the ovaries, the ncRNAs of unknown function were found not ubiquitously expressed.

**Conclusion:**

Our results expand the repertoire of coding/non-coding transcripts derived from the gene encoding Drosophila pseudouridine synthase. This gene exhibits a complex and interlaced organization, and its genetic information may be expressed as different protein subforms and/or ncRNAs that may potentially contribute to its biological functions.

## Background

H/ACA ribonucleoprotein particles (RNP) in eukaryotes consist of four highly conserved core proteins and one molecule of H/ACA small nucleolar RNA (snoRNA), and most of them direct pseudouridylation of target RNAs at specific sites (reviewed in [1,2]). In this process, one of the core proteins acts as a pseudouridine synthase, while the H/ACA snoRNA selects the residues to be isomerized via specific base-pairing. Proteins catalyzing the conversion of uridines to pseudouridines belong to a highly conserved family, well-characterized examples of which include Archaea, yeast and trypanosome Cfb5p [3-5], Drosophila MFL/NOP60B [6,7], rat NAP57 [8], and mouse and human dyskerin [9]. In eukaryotes, these proteins accumulate in the nucleolus and participate in various cellular functions including processing and modification of ribosomal RNA (rRNA) and maintenance of telomere integrity in mammals (reviewed in [10,11]). Genetic depletion experiments in different organisms have invariably shown that these proteins are essential for viability [4,7,12,13], indicating that they have important biological roles. The finding that human dyskerin is involved in two congenital diseases further supports this notion. Mutations in these proteins are responsible for X-linked dyskeratosis congenita (DC) [9] and for Hoyeraal-Hreidarsson syndrome, now recognized as a severe DC allelic variant [14]. Functional conservation of pseudouridine synthases is so remarkable that the archaeal aCbf5p protein has recently been shown to assemble efficiently with a yeast H/ACA snoRNP core component, Nop10, and with human telomerase RNA, which has a H/ACA box motif [15]. The biological role of RNA pseudouridylation is still debated. It has been suggested that it contributes to rRNA folding, rRNP assembly and ribosomal subunit assembly. Subtle enhancing of ribosomal functions such as codon recognition has also been proposed [16]. Remarkably, recent data indicate that mutations in mammalian dyskerin impair translation from IRES (Internal Ribosomal Entry Site) elements, thus specifically affecting cap-independent translation of a subset of mRNAs [17]. However, the role of H/ACA snoRNPs extends beyond ribosome biogenesis. In fact, although rRNA is the most common modification target, spliceosomal snRNAs or tRNAs can also be modified [1,2]. Furthermore, "orphan" snoRNAs that lack complementarity with rRNA or snRNAs have also been described, and it is plausible that they target cellular RNAs that remain unidentified. Since proteins of the Cbf5p family are essential for biogenesis and accumulation of H/ACA snoRNAs, mutations in them might trigger diverse effects. For example, different mutations of human dyskerin have been associated with reduced levels of distinct subsets of H/ACA snoRNAs [18], raising the possibility that some pathological aspects of DC may be related to the particular functions of the specifically-affected forms. Indeed, the repertoire of functions attributed to members of the *Cbf5 *family is wide and continually increasing; these genes may be involved in many biological processes. In yeast, Cbf5p was first described as a low-affinity centromeric DNA binding protein [19]. Subsequently, depletion of Cbf5p was shown to cause nucleolar fragmentation and to disrupt the nucleolar localisation of tRNA [20]. In mice, *DKC1 *alleles carrying hypomorphic mutations led with very high frequency to tumour development, indicating that the gene acts as a potent oncosuppressor [21]. A novel role in snoRNA metabolism has recently been reported for the yeast and mammalian proteins: they are directly involved in the early steps of H/ACA snoRNP assembly, since they are cotranscriptionally recruited at the 3' end of the nascent H/ACA snoRNAs [22-24]. In trypanosomes, RNAi-induced silencing of *Cbf5 *has been shown not only to eliminate pseudouridylation on the spliced leader RNA (SL RNA), but also to abolish its modification at the fourth cap-4 nucleotide [5]. As a result of defects in the SL RNA and decreased modification of the U small nuclear RNAs, trans-splicing was inhibited at the first step of the reaction.

Considering the wide range of biological effects directed by members of the *Cbf5 *gene family, it is plausible that this functional complexity might rely, at least in part, on the production of multiple transcripts with different properties. Since a complex expression pattern is often observed for multifunctional genes in higher eukaryotes, we planned to analyse the molecular organization and transcriptional activity of the Drosophila orthologue in greater detail, with the aim of better defining its coding properties and shedding new light on its complex functions. In previous studies, we isolated the gene encoding Drosophila pseoudouridine synthase, called *minifly *(*mfl; *also called *Nop60b*) [6,7], and showed that the use of alternative 3' UTRs resulted in two main mRNAs, 1.8 and 2.0 kb in length. These two species had different expression profiles, with the 1.8 kb mRNA constitutively expressed in both sexes throughout the life cycle, and the 2.0 kb species mainly expressed in females and maternally transmitted to the developing embryos [7]. However, the two transcripts had identical protein coding potentials and differed only in their 3' untranslated regions (3'UTRs). As with genes encoding proteins involved in the synthesis, structure or function of the translational apparatus, *mfl *was shown to belong to the 5' TOP family (Tract Of Polypyrimidines) [7,25]. Members of this family share a C residue at the +1 position followed by a 5–15 nt polypyrimidine tract in a short 5' non-translated region; their mRNAs typically show growth-dependent translational regulation [26]. We also showed that *mfl *hosts a snoRNA gene of the H/ACA class within one of its introns. This gene, named *snoH1*, directs pseudouridylation of Drosophila 18S rRNA at position U1820 [7], modification of which residue is conserved from yeast to man.

In this paper we report that *snoH1 *is just one member of a variegated cluster of small ncRNA genes hosted within *mfl *introns, revealing that this gene exhibits a much more complex overlapping coding/noncoding architecture than previously suspected. In addition, we show that *mfl *produces two novel coding transcripts. While one of these mRNAs is characterized only by an extended 3'UTR, the second is alternatively spliced, accumulates mainly in females, and encodes a rare variant protein that is predicted to have distinct functional properties. So far, *mfl *is the only eukaryotic pseudouridine synthase gene for which multiple mRNAs have been reported, and the description of the variant protein and the various ncRNAs it can encode may provide useful insights into the various functions of other members of this conserved family.

## Results

### Identification of two novel *mfl *coding transcripts

**Figure 1:**
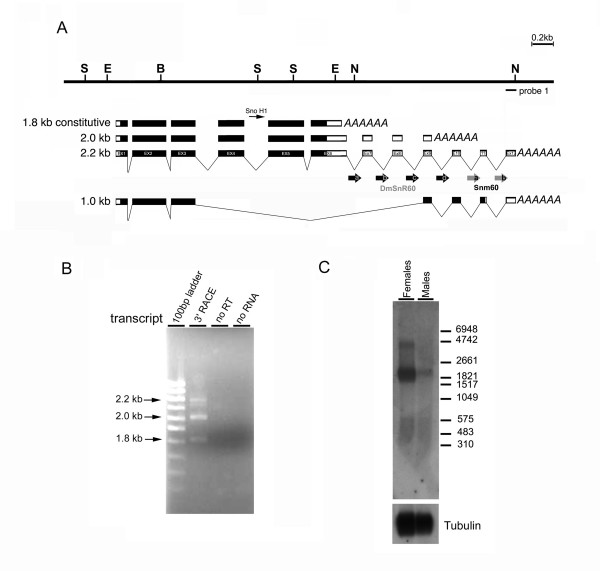
**Molecular structure of *mfl *mRNAs**. (A) Restriction map of the genomic region encompassing the *minifly *gene (S, *Sal*I; E, *Eco*RI; B, *Bam*HI; N: *Not*I) [GenBank: AF 097634]. Below, organization of the previously-described 1.8 [GenBank: AF017230] and 2.0 kb [GenBank: AF089837] *mfl *mRNAs [7], compared with that of the newly identified 2.2 [GenBank: DQ857345] and 1.0 kb mRNA species [GenBank: DQ857346]; note that subsequent releases of the Drosophila genome sequence have revealed that a small intron splits the formerly-designated exon 8 [7] into two moieties, currently indicated as exon 8 and 9. Exonic regions spanned by *mfl *ORFs are depicted in black. The positions of the intron-encoded H/ACA snoRNA *H1 *gene [7] and of the *DmSnR60 *and *snm60 *isoforms are also shown. (B) The products of the 3'RACE reactions were separated on 2% agarose gels and visualised by ethidium bromide staining. Lane 1, GeneRuler 100 bp DNA ladder (MBI Fermentas). Lane 2, products obtained after amplification with a forward primer derived from exon 5, in combination with the oligo-adaptor reverse primer. Specific fragments of about 800, 650 and 400 bp were obtained; each fragment represents the specific 3' end of a different *mfl *mRNA, the length of which (in kb) is indicated on the left. Lanes 2 and 3 show negative controls in which no reverse transcriptase or no input RNA were added to the reaction. (C) Northern blot analysis of poly(A)+ RNA extracted from male and female adult flies with a genomic probe derived from exon 12 (probe1: see the genomic map for position). This probe specifically detects the novel 2.2 kb mRNA, most abundant in females, and a large transcript of about 4.4 kb of which the structure remains to be defined. The amount of RNA loaded on each lane was checked by hybridization with a probe derived from *αTub84B*. The RNA marker I (Roche) was utilised (on the right).

In previous primer-extension experiments we detected and mapped a single *mfl *transcription start site [7] (see Fig.1A). We also noticed that a very short upstream region of about 300 bp separates *mfl *from the close, divergently-transcribed gene *mrp17*. On the basis of these observations, we considered that further transcript heterogeneity is more likely to derive from the alternative 3' ends. Therefore, the transcriptional activity of the gene was first re-examined by 3'-RACE (Rapid Amplification of cDNA Ends). Based on the structures of the previously-described 1.8 and 2.0 kb mRNAs (depicted in Fig. 1A) [Gen Bank: AF017230, AF089837], internal primers were derived from the sequence of exons 5, 6, 8 and 9 (see Methods for sequences) and used to search for additional poly(A) sites, using poly(A)+ RNA extracted from male and female adult flies as template. In addition to the two expected bands, a longer product was obtained in each 3'-RACE reaction, revealing the presence of a poly(A) site further downstream (Fig. 1B). These additional products were markedly more abundant in the female RNA preparation, implying a sex-bias in the use of this novel 3' site. Subsequent nucleotide analyses of these amplification products confirmed that *mfl *encodes a third mRNA that differs from the 1.8 and 2.0 kb species only in the presence of three additional exons in a longer 3'UTR (Fig. 1A) [Gen Bank: DQ857345]. Northern analysis of the poly(A)+ RNA preparations probed with the novel downstream exons revealed a transcript of about 2.2 kb, expressed mainly in females (Fig. 1C). This is the expected length for an mRNA species starting at the previously-mapped 5' site and terminating at the novel 3' end, further confirming that the 1.8, 2.0 and 2.2 kb mRNAs are distinguished solely by 3'-end heterogeneity. We then searched for *cis*-acting control elements affecting mRNA stability, location or translation within these overlapping 3'UTR sequences. However, no functional motif currently annotated in the UTResource database [27] was identified in any of these regions. Further experiments will therefore be required to establish whether these sequences include still-unidentified regulatory motifs. Strikingly, Northern analyses of poly(A)+ RNA revealed that a large polyadenylated transcript of about 4.4 kb also derives from this downstream region (Fig. 1C). This species had already been detected in adult female or embryonic RNA preparations by more upstream *mfl *genomic probes [7], but previous attempts to isolate cDNA clones representing this form proved unsuccessful even after extensive screening of various cDNA libraries. Since in the present set of experiments we again failed to identify any product representing this RNA, its structure remains elusive, so we cannot exclude the possibility that this species represents a variant transcript extending much further, or an unprocessed RNA precursor.

**Figure 2:**
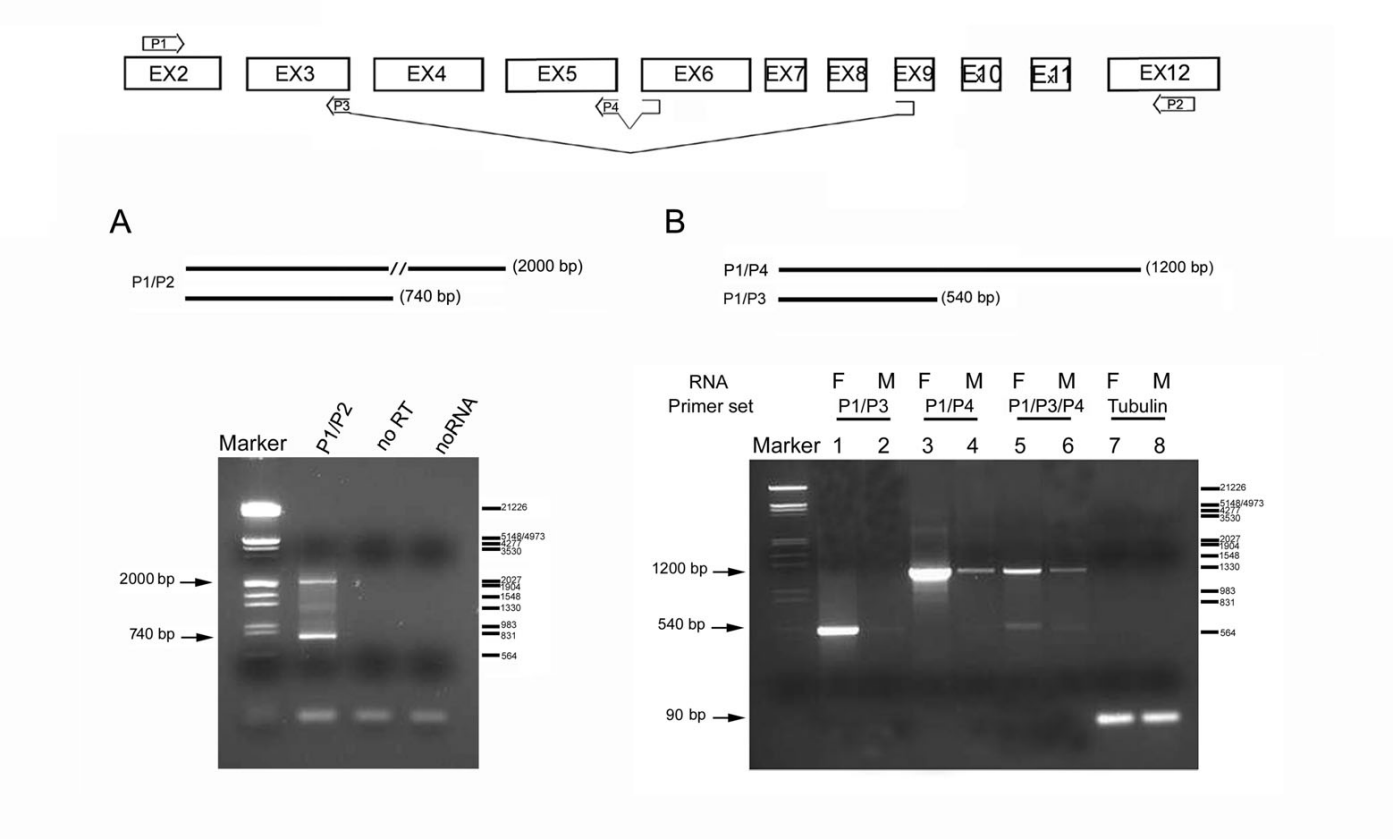
***mfl *encodes a novel alternatively-spliced mRNA**. At the top, a schematic drawing of the splicing patterns generating *mfl *mRNAs; the position and structure of the primers utilised in RT-PCR assays are indicated. HindIII-digested λ DNA was used as marker; base pairs are indicated on the right. (A) RT-PCR performed with poly(A)+RNA isolated from adult females and an exon 2–12 primer pair (P1/P2). The 2000 bp fragment is specific for the 2.2 kb mRNA, while the 740 bp fragment derives from the 1.0 kb alternatively-spliced subform. (B) RT-PCR was performed with poly(A)+RNA isolated from male (M) and female (F) adult flies. Female or male samples were amplified using the same forward primer (P1, derived from exon 2) in combination with a reverse primer spanning the alternative 3–9 exon junction (P3; lanes 1–2) or the canonical 5–6 exon junction (P4; lanes 3–4); in lanes 5–6, the three primers were added to the same reaction. The 1200 bp fragment represents all three transcripts generated by the canonical splicing pattern, while the 540 bp fragment derives specifically from the 1.0 kb mRNA. In lanes 7–8, a 90 bp fragment representating the *αTub84B *transcript was amplified as internal control of the quantity of RNA.

We next tried to connect the novel 3' exon cluster with more upstream exons by RT-PCR, with the aim of detecting alternative splicing events within the coding sequence of the gene. For this purpose, we used poly(A)+ RNA from male and female adult flies as template, and various combinations of forward primers positioned inside the coding region (over exons 2, 5 and 6) with reverse primers positioned along the 2.2 kb-specific 3'UTR exons (exons 10, 11 and 12; see Methods). In each combination, primers derived from exons 5 or 6 always yielded a single amplification product, markedly more abundant in females; sequence analysis invariantly confirmed the structure of the 2.2 kb mRNA previously obtained by 3'-RACE. In contrast, two types of products were always obtained when a forward primer from exon 2, in each combination of reverse primers, was used to reverse-amplify female RNA. In addition to the expected band, a minor product of smaller size was noticed in each reaction. As an example, the result of an exon 2–12 RT-PCR amplification from female RNA is reported in Fig. 2A. In this case, in addition to the 2 kb product expected from the 2.2 kb mRNA, an additional product of 740 bp was observed.

Sequence analysis of the additional shorter products obtained in this set of experiments revealed an alternative splicing event that joins the 5' splice donor of exon 3 to the 3' splice acceptor of exon 9. To confirm the presence of this spliced mRNA, the same 5' primer was used in combination with either a 3' primer spanning the 3–9 exon junction, able to detect only the alternatively spliced RNA subform, or one spanning the 5–6 exon junction, able to detect the whole set of transcripts generated by the canonical splicing pattern comprising the 1.8 kb constitutive mRNA and the 2.0 and 2.2 kb maternal species. Both primer pairs yielded positive amplification of a fragment with the expected size; moreover, both products were obtained at a significantly higher level in females, indicating that, as previously described for the 2.0 kb [7] and also for the 2.2 kb species described above, the variant mRNA exhibits a sex-preferential expression profile (Fig. 2B).

**Figure 3:**
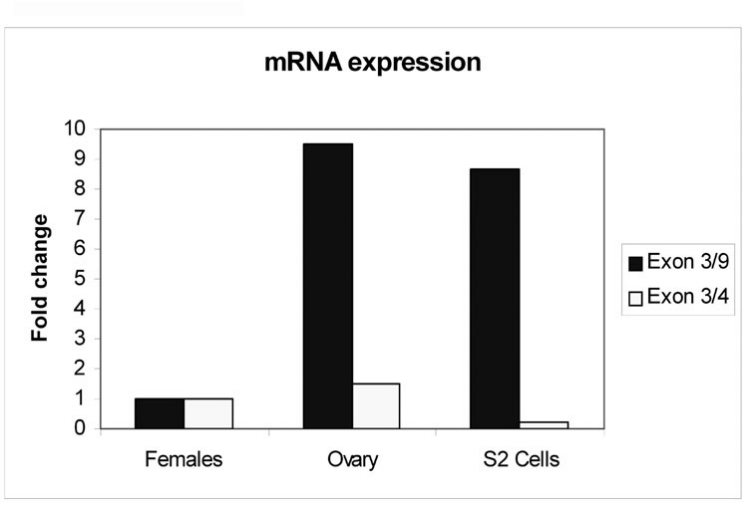
**Relative abundance of alternatively- and canonically-spliced *mfl *mRNA subforms**. The abundances of the alternatively- and canonically- spliced *mfl *mRNAs were measured by quantitative real-time RT-PCR in poly(A)+ RNA from adult Drosophila females, manually-dissected ovaries and cultured S2 cells. Three different RNA extractions were examined for each sample, and each reaction was performed in triplicate. Data were normalized to *αTub84B *expression and are presented relative to the female sample; they represent three independent experiments.

A full-length cDNA clone representing this novel mRNA was obtained by combining 5' and 3' RACE experiments. Gene-specific primers were designed to amplify the 5' end of the transcript and to give fragments partially overlapping with the 3'-RACE products (see Methods). A short overlapping sequence in the 5' and 3' products allowed us to construct a virtually full length cDNA by restriction digestion at the common *Bam*H1site. The fragment obtained was about 1 kb and its nucleotide sequence [GenBank: DQ857346] indicated that the novel mRNA starts at the unique, previously-mapped transcription start site [7]. Intriguingly, this transcript is characterized by the skipping of five internal exons and the absence of any internal stop codon, indicating that it may encode a novel protein subform (Fig. 1A). Given that the 1.0 kb mRNA species was barely detected in the Northern analyses of poly(A)+ RNA preparations, we used quantitative real-time PCR to check its relative abundance in the poly(A)+ RNA preparations obtained from various sources: adult females, manually dissected adult ovaries and cultured Schneider 2 (S2) Drosophila cells. The relative abundances of the variant and canonically-spliced mRNAs (the 1.8, 2.0 and 2.2 kb subforms) were compared among the three samples after normalization against *αTub84B *expression. The 1.0 kb mRNA was significantly more abundant in ovaries than in whole adult females and its accumulation was at least nine-fold greater; the canonically-spliced mRNA was also slightly enriched in this organ. In proliferating S2 cells, the alternatively-spliced species was markedly more abundant, whereas the level of the canonically-spliced mRNAs was slightly reduced (Fig. 3).

### Structural properties and expression of the MFLα novel protein subform

**Figure 4:**
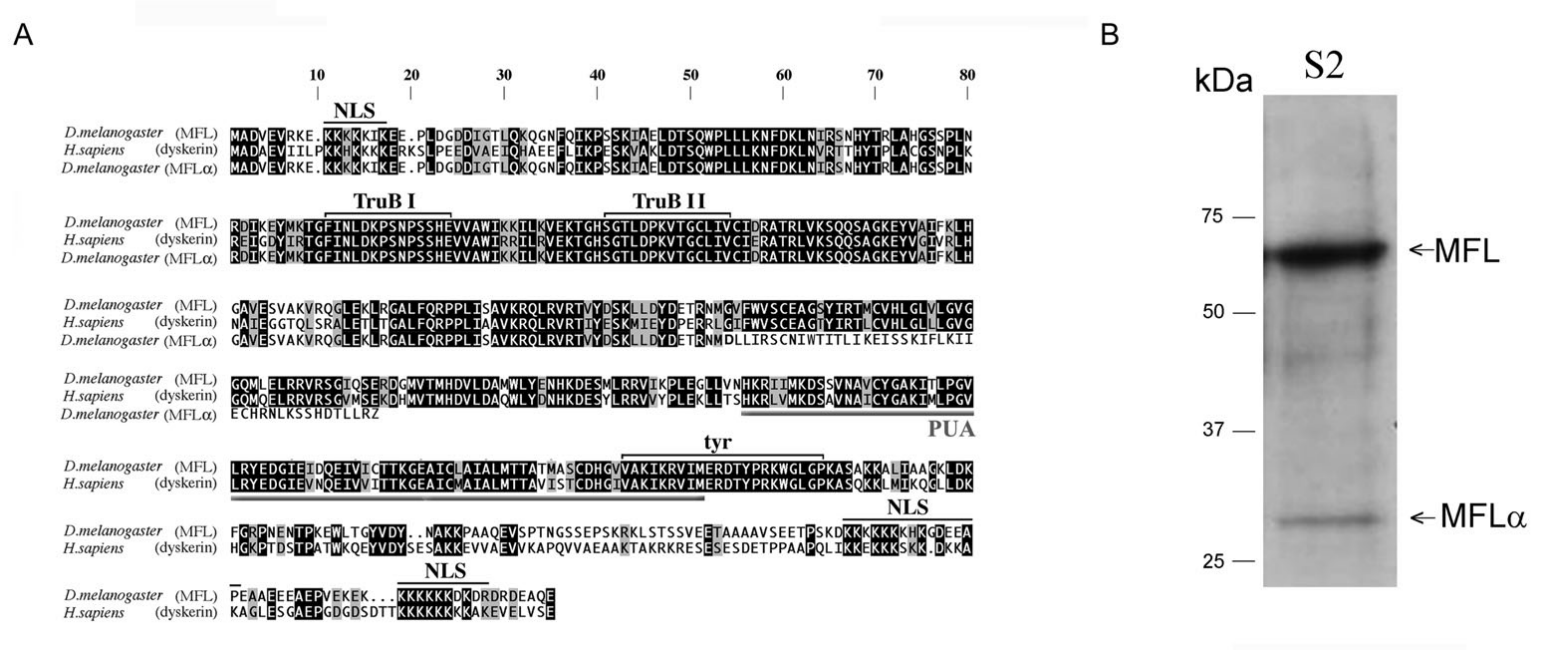
**Amino acid sequence and expression of the novel MFLα protein subform**. (A) Alignment of MFL (top), dyskerin (middle) and MFLα (bottom) amino acid sequences. White letters highlight identical amino acids on a black background, and different but conserved amino acids on a grey background; block letters on a white background indicate different and non-conserved residues. Lines above or below the sequences indicate putative functional domains. NLS: nuclear localisation signal; TruB I, Trub II: regions having homology with members of the bacterial tRNA pseudouridine synthase family; tyr: tyrosine domain containing a putative uracil binding pocket; PUA: a conserved RNA binding domain observed in archaeal, bacterial and eukaryotic RNA modifying proteins. (B) Western blot analysis of extracts from S2 cells. Affinity-purified rabbit polyclonal antibodies recognizing both MFL and MFLα were utilised (see Methods). A major band corresponding to the MFL subform (56 kDa) and a minor band corresponding to the MFLα subform (29 kDa) were detected. Positions of the protein ladder (Precision Plus Prestained Protein Standard Dual Colour; Biorad Laboratories) are shown on the left.

The rare alternatively-spliced *mfl *mRNA is predicted to encode a variant protein of 254 amino acids, with a molecular mass of 28.9 kDa, which we named MFLα. The amino acid sequence of this variant protein is shown in Fig. 4A, aligned below that of MFL, identically encoded by the 1.8, 2.0 and 2.2 mRNAs, and that of human dyskerin. MFLα fully overlaps the MFL sequence at its amino-terminal region, where both proteins exhibit an identical tract of 211 aa that includes the N-terminal nuclear location signal (NLS), and the two highly-conserved TruBI and TruBII motifs that share homology with bacterial and yeast tRNA pseudouridine synthases and are directly involved in the pseudouridylation process. However, MFLα exhibits a unique C-terminal tract of 43 residues that shows no significant homology with any known functional motif. Instead, this region replaces a large carboxy-terminal portion of the MFL protein that includes at least three domains for which a functional role has been proposed. The first missing motif corresponds to the PUA domain, an RNA binding motif observed in several families of archaeal, bacterial and eukaryotic RNA-modifying proteins including pseudouridine synthases and rRNA methylases. This domain has also been found in bacterial and yeast glutamate kinases, as well as in families of eukaryotic proteins that are thought to act as translation factors [28]. Intriguingly, it has recently been shown to play a crucial role in archaeal snoRNP assembly, and to be necessary for aCbf5p binding to guide RNAs [29]. The second missing domain is represented by a block of more than twenty residues with a central tyrosine (tyr) that is identical in MFL and human dyskerin and is highly related to the uracil-binding pocket in uracil-DNA glycosylases [7]. Finally, MFLα also lacks a highly-charged lysine-rich carboxy-terminal region that contains an overlapping bipartite NLS, raising the possibility that this variant protein may have a different subcellular distribution, or that it is less tightly retained in the nuclei.

Collectively, these distinctive features suggest that MFLα may have a distinct role in at least some of the essential cellular activities of *mfl*. As a preliminary step to investigating this possibility, we attempted to assess the effective *in vivo *accumulation of this rare subform. On the basis of the observations indicating a higher abundance of the 1.0 kb mRNA in S2 cells, we selected this source for checking the presence of MFLα protein by Western blotting. Rabbit polyclonal antibodies were raised against two peptides, both included in the common N-terminal region of MFL/MFLα (see Methods), and protein extracts from S2 cells were subjected to Western analysis with these antibodies. In addition to the canonical MFL protein, a less abundant band of the molecular weight expected for MFLα was detected (Fig. 4B), indicating that the alternatively-spliced mRNA may encode a variant protein.

### A cluster of small ncRNA genes is intron-encoded at the *mfl *variable 3' UTRs

**Figure 5:**
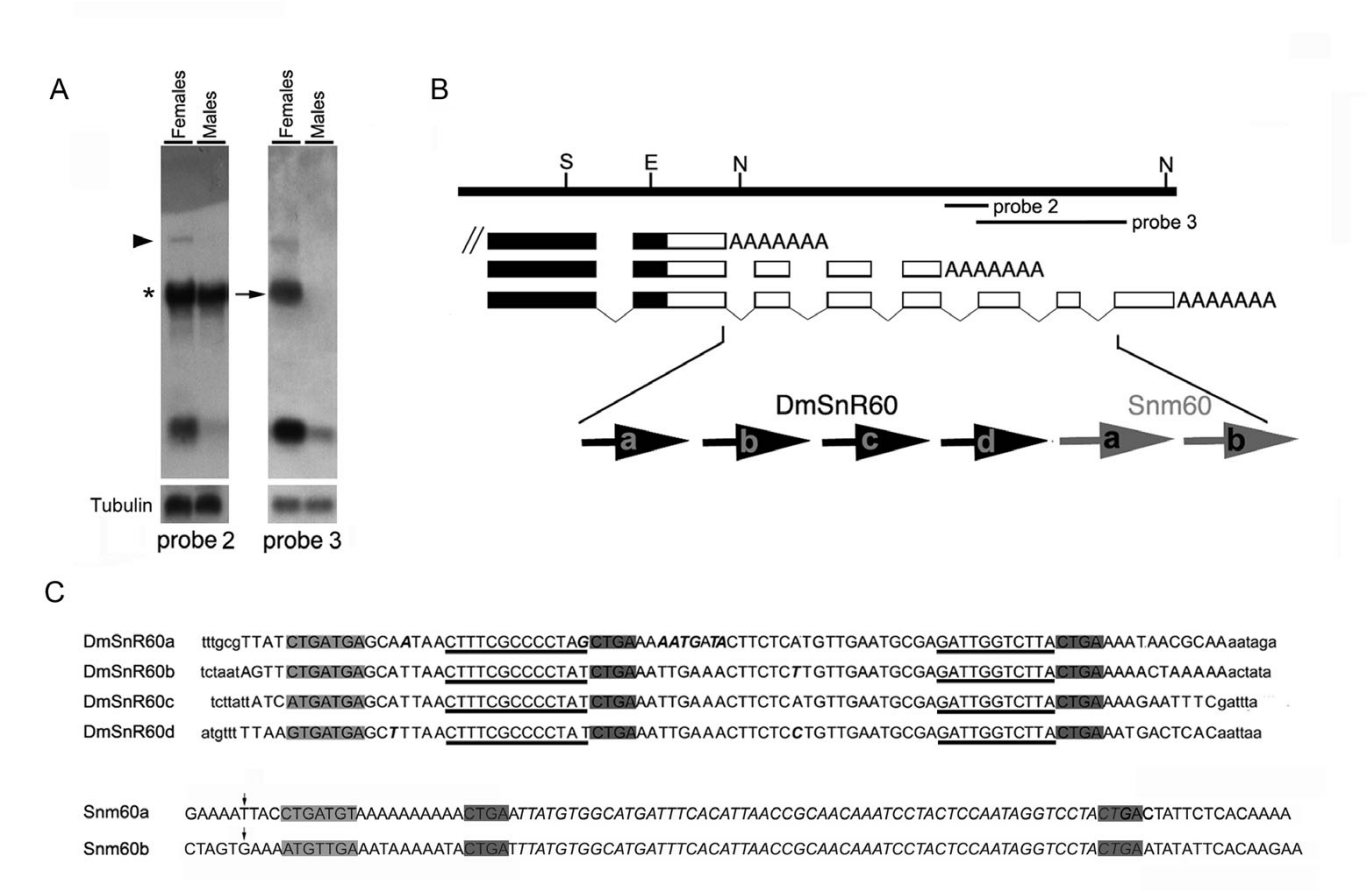
**An intronic cluster of ncRNA genes maps at the variable *mfl *3' UTRs**. (A) Northern blot analysis of total RNA from male and female adult flies with genomic probes derived from the *mfl *3' region (see B for genomic position). Probes 2 and 3 both detect a large transcript of about 4.4 kb (marked by the triangle) and small RNAs of about 100 nt. Note that probe 2, derived from intron 9, strongly cross-hybridizes with rRNA (asterisk on the left) because of bipartite *DmSnR60 *complementarity to 28S sequences. Since Drosophila 28S rRNA is processed into two fragments that migrate in a similar manner to the 18S rRNA (2.0 kb), cross-reaction labels a band with a mobility similar to that of the *mfl *2.2 kb mRNA (indicated by the arrow), which is specifically recognized by probe 3. (B) Genomic organization of the cluster of small ncRNA genes intron-encoded at the *mfl *3'UTRs; the four copies of the *DmSnR60 *C/D box snoRNA gene (*a, b, c, d*) and the two copies of the *snm60 *(*a, b*) exhibit a one gene-per intron organization. (C) Nucleotide sequences of *DmSnR60 *and *snm60 *isoforms. Within *DmSnR60 *sequences (capital letters; flanking sequences are indicated as lower case letters), dark-shaded regions indicate the D and D' boxes, while the 5'-terminal C box is grey-shaded. The D and D' antisenses able to target the Drosophila 28S rRNA are underlined. Positions of nucleotide polymorphisms among the *DmSnR60 *isoforms are indicated in italics. At the bottom, nucleotide sequences of the *snm60 a *and *b *isoforms [GenBank: DQ142641; DQ142642]. The vertical arrows mark the position of the 5' end of the products mapped by primer-extension (see Methods). The putative D and D' boxes are dark-shaded, the 5'-terminal C box is grey-shaded and the internal segment of perfect identity shared by the two *snm60 *subforms is in italics.

To check *mfl *transcription further, we performed Northern blotting of total RNA preparations using genomic probes spanning the variable *mfl *3' ends. The results showed that a set of small RNA molecules, about 100 nt long and highly abundant in females, also derive from this region. To map these molecules in greater detail, shorter probes having either exonic or intronic localisation were used to analyse RNA preparations from adult flies of both sexes (Fig. 5A). Probes derived from introns 6, 7, 8 and 9 all detected the 4.4 kb species (marked by a triangle). They also detected a radioactive signal of about 85 nt, as estimated by carefully assessing the RNA length on 6% denaturing polyacrylamide gels and by mapping the 5' end by primer extension analysis (data not shown; see Methods). Inspection of the *mfl *3' genomic sequence [GenBank: AF097634] indicated that introns 6, 7, 8 and 9 each host a copy of *DmSnR60 *(Fig. 5B–C), a snoRNA gene of the C/D family previously identified in the course of a genome-wide computational search [30]. The strong cross-hybridization with rRNA shown by these intronic probes (Fig. 5A, asterisk on the left) is essentially explained by their perfect complementarity to the 28S rRNA molecule, which is recognized by long D and D' antisense elements (see below). As shown in the figure, probes spanning introns 10 and 11 detected RNA molecules estimated at 100 nt, again on the basis of 6% denaturing polyacrylamide gel electrophoresis and primer extension analysis of the 5' end, in addition to the 4.4 and 2.2 kb mRNAs. Nucleotide analysis of introns 10 and 11 revealed that each hosts a copy of a small ncRNA gene, the sequence of which substantially overlaps that of the DmOrC/D_9 a-b molecules [GenBank: AY805216] previously described by Huang et al. [31]. These molecules exhibit canonical D and D' boxes (Fig. 5A–C) but have a degenerate C motif with a sequence varying between the two tandemly-repeated isoforms. The biological functions, if any, of these molecules remained to be firmly established, so they were classified as orphan C/D snoRNAs [31]. Indeed, we noticed that they lack a nucleolar- or Cajal body- specific location (see below), so in accordance with widely-accepted nomenclature [32], we refer to them as small non-messenger RNAs derived from polytene region 60 (*snm60*). The two copies of *snm60 *(*a, b*) [GenBank: DQ142641 and DQ142642] share 85% overall sequence conservation and exhibit a 59 bp internal segment of perfect identity (Fig. 5C). As judged by Southern blotting (data not shown) and a computational search on the genome sequence, no other *snm60 *copy is present elsewhere in Drosophila.

Organization of the ncRNA gene cluster mapped at the *mfl *3'-UTRs conformed to the one-gene-per-intron rule widely observed in animal genomes. Moreover, we noticed that the tandemly-clustered *DmSnR60 *genes are all located about 70 nt upstream the 3' splice site of their host introns. In vertebrates, a position about 70 nt upstream the 3' splice site is reportedly optimal for expression of intronic C/D box snoRNAs, since this distance may furnish optimal synergy with splicing, favouring C/D box snoRNP assembly [33]. The conserved position of *DmSnR60 *isoforms indicates that the rule observed by Hirose and coworkers [33] may also be operative in invertebrates. *DmSnR60 *genes, first described by our group [30], were reported in a subsequent genome-wide analysis to be generated, along with DmOrC/D_9 a-b molecules, by introns of a long, polyadenylated non-coding host transcript named dUhg 6 [31]. However, identification of the additional *mfl *poly(A)+ site in the present study provides clear evidence that *DmSnR60 *and DmOrC/D_9/*snm60 *copies are all intron-encoded by this protein-coding gene.

An intriguing functional consequence of the *mfl *coding/non-coding arrangement is that pre-mRNAs ending at different alternative poly (A) sites can release distinct sets of nested ncRNAs. In fact, a pre-mRNA molecule ending at the first poly(A) site can exclusively produce the H/ACA *snoRNA H1*, while one ending at the middle site may also release three *DmSnR60 *isoforms (*a, b, c*). A pre-mRNA terminating at the most downstream site may instead produce three different types of small ncRNAs, including *snoH1 *and all the *DmSnR60 *(*a-d*) and *snm60 *(*a-b*)isoforms. Strikingly, the ability to release *snm60 *molecules is restricted to *mfl *transcripts ending at the last poly (A) site, so that production of these ncRNAs is predicted to be coupled with that of the 2.2 and 1.0 kb mRNAs. In conclusion, alternative 3'-ends allow *mfl *to produce not only two distinct protein subforms, but also different ncRNAs that may potentially contribute to its biological functions.

### Expression and function of *mfl *intron-encoded ncRNAs

**Figure 6:**
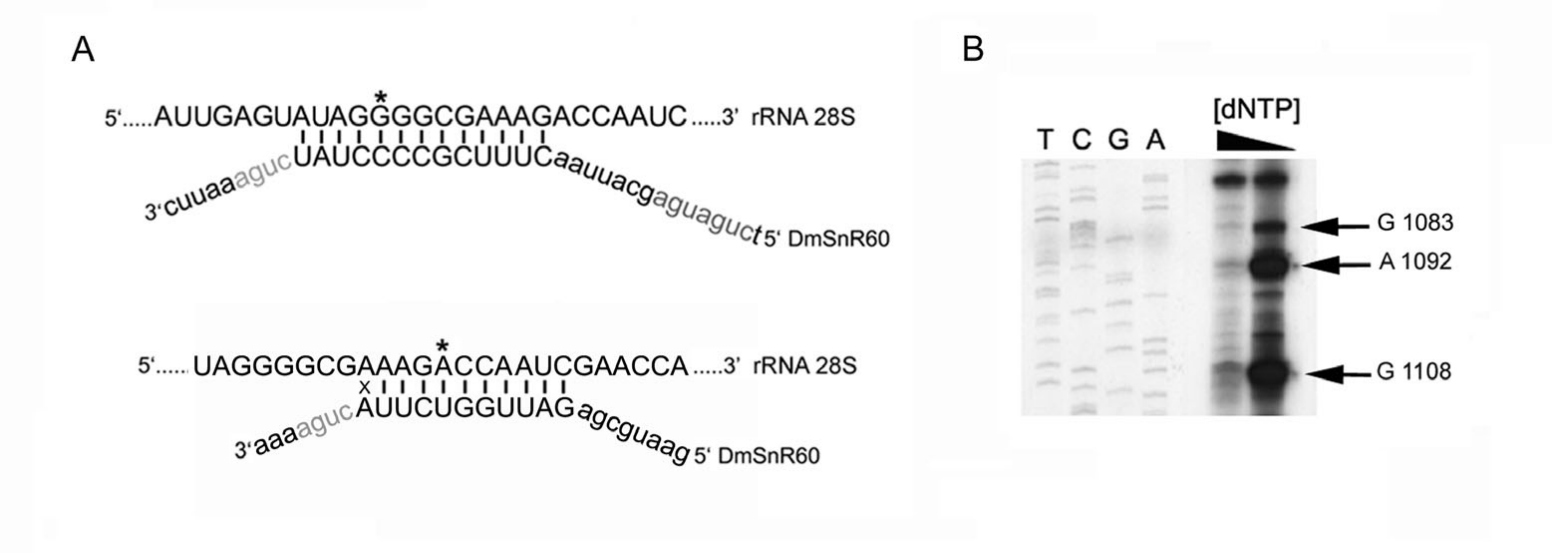
**Functional roles of *DmSnR60 *molecules**. (A) Base-pairing interactions between *DmSnR60 *and *Drosophila *28S rRNA sequences. The upper strand represents the rRNA sequence; the G1083 and the A1092 residues selected for methylation by D' and D antisense elements are marked by the asterisk. (B) Reverse transcription at low dNTP concentration of the 28S Drosophila rRNA region, including the G1083 and the A1092 methylation sites; on the left, lanes T, C, G and A are dideoxy sequence reactions performed using the primer utilised for reverse transcription and a plasmid carrying the *Drosophila *28S rRNA gene. One additional 2'-O-methylation, at position Gm1108 of 28S rRNA, is detected in this experiment. This methylation has not yet been described in other organisms and may be specific to Drosophila rRNA.

All the *DmSnR60 *isoforms (named *a, b, c, d*) possess canonical C (5'-UGAUGA-3'), D and D' (5'-CUGA-3') boxes, suggesting that they encode bifunctional snoRNAs of the C/D family (Fig. 5C). Long tracts of perfect complementarity to Drosophila 28S rRNA were found upstream of both the D and D' boxes [30,31]. Since C/D snoRNAs invariably select the nucleotide positioned 5 base pairs upstream of the D/D' box for methylation [1,2], these antisense elements were predicted to modify, respectively, the G1083 and Am1092 residues (see Fig. 6B), two methylation sites conserved between yeast and vertebrates. In fact, methylation at the G1083-equivalent residue is known to be guided by yeast snR60 and mammalian U80 snoRNAs, while Am1092 modification is directed by yeast snR84 and by a still-unidentified mammalian snoRNA [34]. Methylated residues have not yet been experimentally mapped on Drosophila rRNA, so we attempted to detect effective modification at the predicted sites by reverse transcription of the specific rRNA sequence at low dNTP concentration, as described by Maden [35]. When this method is used, a specific reverse transcription stop occurs on (and/or one nucleotide before) a 2'-O-methylated nucleotide at low, but not at elevated, dNTP concentrations. As shown in Fig 6B, this experiment confirmed the presence of methylated nucleotides at both predicted positions. Strikingly, one additional 2'-O-methylation was detected, at position Gm1108 of 28S rRNA (Fig. 6B). Modification at this site has not yet been described in other organisms and may be specific to Drosophila rRNA. Since no other Drosophila snoRNA that may be able to methylate the G1083 and the Am1092 residues specifically has so far been described, modification of these sites strongly supports the functional role of the *DmSnR60 *molecules as rRNA methylation guides. In contrast, the function of the *snm60 *molecules is hard to guess, and further experiments are required.

**Figure 7:**
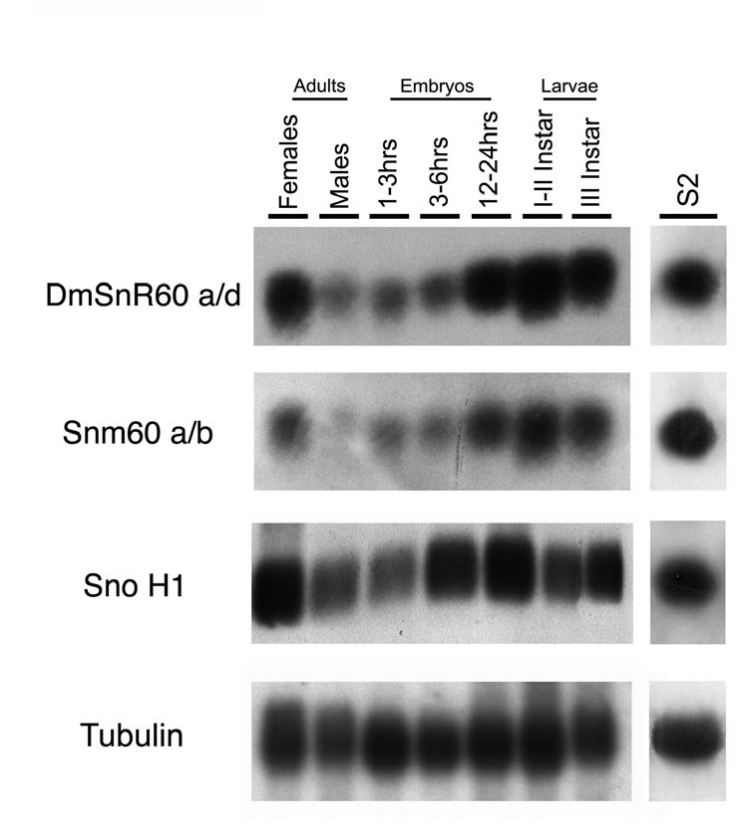
**Developmental expression of *mfl *intron-encoded ncRNAs**. Total RNA extracted at various developmental stages of the Drosophila life cycle was hybridized to probes specific to *DmSnR60, snm60 *and *snoH1*. On the right, Northern blot analysis of total RNA extracted from the Drosophila S2 cell line. The amount of RNA loaded on each lane was checked by hybridization with a probe derived from Drosophila *αTub84B*.

To characterize further the small ncRNAs originating from the *mfl *3' region, a panel of total RNA samples extracted from various stages of Drosophila development or from the S2 cell line was subjected to Northern blot analysis with probes specific for *snoH1, DmSnR60 *or *snm60*. These intron-encoded ncRNA genes were all actively expressed in S2 cells, and throughout the Drosophila life cycle they exhibited a very similar expression pattern (Fig. 7). As shown in Fig. 7, the ncRNAs accumulate constitutively throughout development, but their expression is quantitatively modulated, reaching the highest levels in young larvae and in adult females. It should be noted that the marked sex-bias in *snoH1 *and *DmSnR60 *expression is quite unusual for snoRNAs. Considering that these snoRNAs are devoted to modifying, respectively, the 18S and 28S rRNAs, it is plausible that the higher levels in females may essentially be due to the higher level of protein synthesis in the ovaries, which in adult females may constitute more than half the body weight.

**Figure 8:**
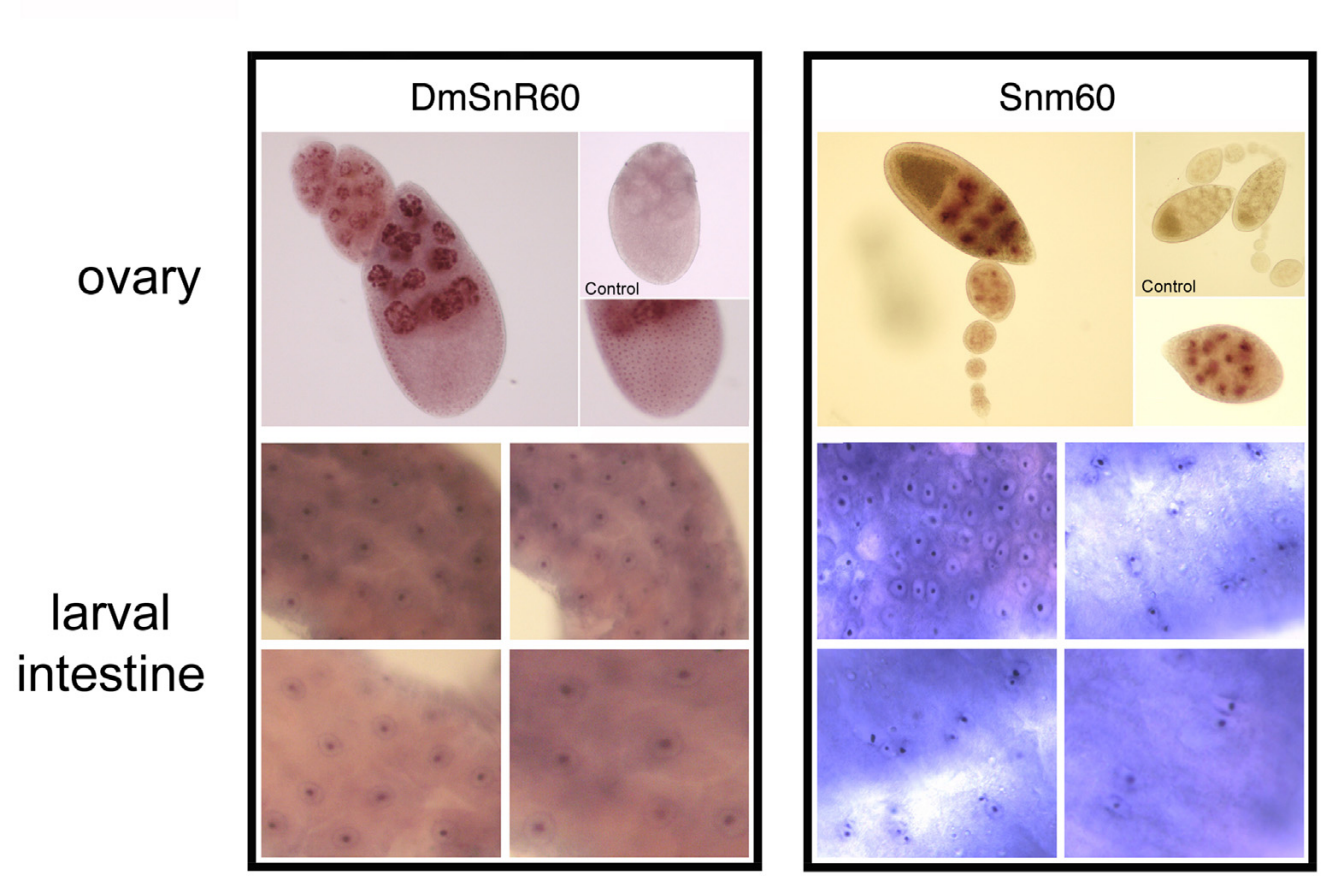
**Cellular location of *DmSnR60 *and *snm60 *molecules**. Top-left panel: whole mount *in situ *hybridization of *DmSnR60 *specific probes to adult ovaries. *DmSnR60 *molecules are detected at all stages of oogenesis, in the nurse and the follicle cells (see enlargement on bottom-right). Analysis of the large polyploid nurse-cell nuclei reveals that the *DmSnR60 *molecules are located in the peripheral nucleolar structures. Hybridization of the sense probe was performed as control (top-right). Bottom-left panel: *in situ *hybridization of *DmSnR60 *probes to larval intestines. Top-right panel: whole mount *in situ *hybridization of *snm60 *specific probes to adult ovaries. *snm60 *molecules accumulate specifically in the nurse cell nuclei from stage 7 of oogenesis, and are not detected in the follicle cells (see also the enlargement at bottom-right). Hybridization of the sense probe was performed as control (top-right). Bottom-right panel: *in situ *hybridazion of *snm60 *probes to larval intestines.

To address the function of *DmSnR60 *and *snm60 *further, we next investigated their intracellular distribution by *in situ *hybridization. Given that these RNAs accumulated at higher levels in larvae and in adult females, we selected the intestine as a larval organ and the ovary as an adult organ for these analyses. When specific digoxigenin-labelled antisense probes were used to analyse whole-mount intestine preparations, the *DmSnR60 *molecules were shown to be expressed in all cells (Fig. 8). Within ovarioles, these molecules were also expressed ubiquitously, being detected at all stages of oogenesis in both the nurse and the follicle cells (Fig. 8, top-left panel). Moreover, *DmSnR60 *molecules accumulate specifically in the nucleoli, as substantiated by observation of the large nuclei of the polyploid nurse cells. These cells develop unusual nucleoli comprising a shell of interconnected fibres around the nuclear periphery [36]. As is evident from the figure, the *DmSnR60 *hybridization signal in the nurse cells is specifically concentrated in these nuclear peripheral structures, as expected for *bona fide *snoRNAs (Fig. 8, top-left panel). In contrast, *snm60 *expression in the ovarioles appeared to be restricted to late oogenesis, starting from stage 7 (Fig. 8, right-top panel); moreover, these RNAs accumulated specifically in the nurse cells, not the follicle cells. Within the nurse cells, the *snm60 *molecules appear to be concentrated in the nucleoplasm but are not located within the nucleoli. These ncRNAs also show an intriguing expression pattern in the larval intestine, where in most microscopic fields they are detected only in subsets of cells that appear to be actively dividing (Fig. 8, bottom-right panel). Most cells expressing *snm60 *RNAs are in fact yet-unseparated daughter cells just exiting from mitosis, or paired cells that plausibly derive from the same mitotic event, raising the possibility that expression of these molecules might be regulated during the cell cycle. An obvious conclusion from these *in situ *experiments is that spatial and temporal expression of *DmSnR60 *and *smn60 *genes are differentially regulated. It is possible that *smn60 *molecules may be independently transcribed from a specific alternative promoter, or that their biogenesis and/or stability may depend on the presence of specific factors. Whatever the case, such fine regulation was unexpected and makes it unlikely that these small RNAs merely represent transcriptional noise.

**Figure 9:**
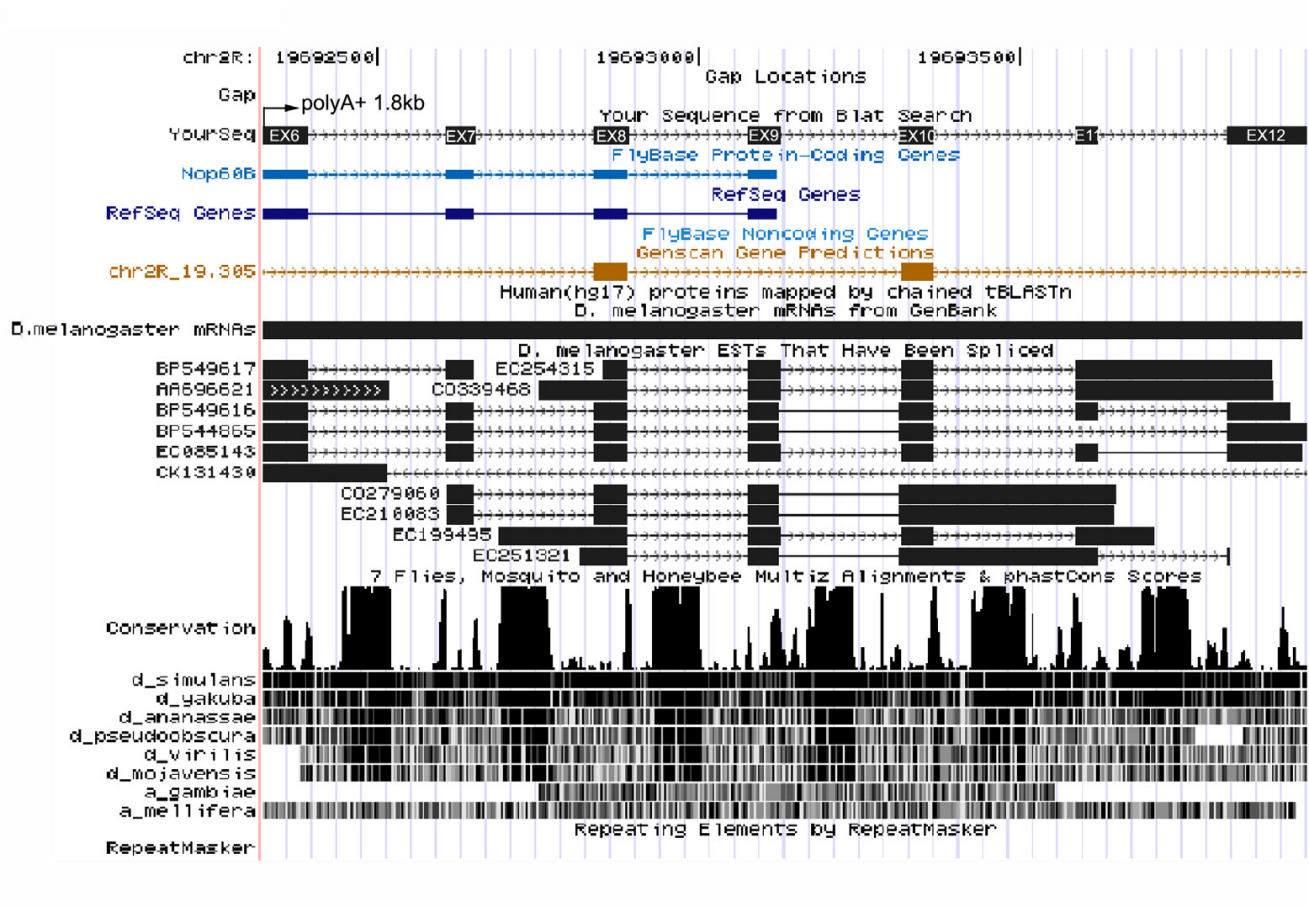
**Screen shot of the UCSC Genome Browser () conservation tracks of the *mfl *3' genomic region**. The region examined, spanning exons 6–12 (black boxes), is shown at the top. The conservation track has two parts: a plot of conservation scores, and beneath it, a display showing where each of the other genomes aligns to the reference sequence (darker shading indicates higher BLASTZ scores; white indicates no alignment). Peaks of cross-species conservation are observed at each 3 'UTR intron, but only sequences of the *DmSnR60 *isoforms are highly conserved among all the annotated genomes of the Drosophila species.

To investigate this aspect further, we checked the phylogenetic conservation of *smn60 *genes by searching for their expression in Drosophila-related species such as *D. yakuba, D. virilis *and *D. ananassae*. By Northern blot experiments, we were able to detect these ncRNAs only in the most closely-related species *D. yakuba *(data not shown), which belongs to the *melanogaster *subgroup. In contrast, the *DmSnR60 *molecules were positively detected in all three species. A comprehensive picture of sequence conservation of the *mfl *3' genomic region is presented in Fig. 9, which shows a diagram of multiple genome alignments obtained by the UCSC Genome Browser [37]. Peaks in the conservation plot can be observed for each of the 3'UTR introns, but strong conservation among all the annotated genomes of the Drosophila species occurs only for *DmSnR60 *isoforms. The weaker phylogenetic conservation displayed by *snm60 *suggests that these genes may have evolved more recently. However, evolutionary conservation may not be a reliable signature for functional ncRNAs, since many regulatory ncRNA evolve quickly and may co-evolve with their functional targets [38].

## Discussion

Production of multiple transcripts with different coding/non-coding properties is known to contribute largely to the complexity of eukaryotic transcriptomes, and analysis of the full range of different transcripts that a gene can encode may provide important insight into its biological functions. Although alternative mRNA subforms have never been described for any member of the *Cbf5/mfl/DKC1 *family, transcripts that have very low abundance, or are expressed in selected cell types or in response to specific stimuli, may have escaped analysis. Indeed, rare products have important roles in many physiological processes and can often trigger crucial responses to developmental or growth stimuli. The detailed analyses of the *mfl *gene reported here show that its molecular organization is much more complex than previously suspected and its coding potential is correspondingly expanded. Indeed, *mfl *is the only pseudouridine synthase gene for which extensive evidence of multiple transcripts has been reported so far. Our results showed that the canonical MFL protein can be encoded by three mRNAs distinguished solely by 3' end heterogeneity, two of them displaying a maternal pattern. These overlapping 3'-UTRs may plausibly play a role in imposing different expression profiles on the *mfl *mRNAs. In this regard, an intriguing recent suggestion is that translation of 5'-TOP mRNAs may become less stringent with increasing 3'-UTR length [39]. Consistent with this notion, it could be surmised that the diverse lengths of 3'-UTRs in the 1.8, 2.0 and 2.2 kb mRNAs may *per se *influence the efficiency of their translation under different growth conditions; moreover, the 1.0 kb mRNA is the species for which the most stringent translational regulation should be expected.

The data reported in this paper also reveal that *mfl *encodes a novel alternatively-spliced protein subform, which represents the first example of a variant protein encoded by any member of the *Cbf5 *gene family. The main distinctive trait of this protein, named MFLα, is its unique C-terminus, where a short tract of 43 amino acids replaces the large carboxy-terminal moiety of the major canonical protein, which includes the overlapping PUA and tyr domains and the bipartite NLS. Given that the PUA domain of archaeal aCbf5p has recently been shown to be essential for binding the guide RNAs [29,40], its lack is predicted to affect this functionally relevant feature, raising the possibility that MFLα may participate in the formation of less efficient, or even inactive, H/ACA snoRNPs. If this were the case, expression of this subform might have an autoregulatory role in *mfl *expression. However, we cannot presently exclude the possibility that its specific C-terminus may allow MFLα to interact with different efficiency or different specificity with distinct protein partners, eventually participating in the assembly of specific snoRNP subtypes. On the other hand, neither can we exclude the possibility that this subform has so far unknown functions, even unrelated to those of the major canonical protein. Indeed, a further relevant distinctive trait of MFLα is the absence of the C-terminal NLS. This might plausibly allow a subtly modified modulation of the rate of nuclear transport, or of nuclear retention, or of the subnuclear distribution of the MFLα subform in response to a various cellular stimuli. In this context, it is intriguing to note that deletion of the C-terminal lysine-rich cluster did not affect the nucleolar location of human dyskerin, but influenced the rate of transport into the nucleus [41], while truncation of the C-terminal basic domain of yeast Cbf5p led to delay at the G2/M phase of the cell cycle [19].

Finally, we noticed that the MFL C-terminal moiety deleted in MFLα is particularly rich in putative phosphorylation sites. Since many cellular processes are controlled in a phosphorylation-and cell cycle-dependent manner, including protein synthesis and cell division, it is conceivable that MFL and MFLα may respond in different ways to various growth conditions and cellular signals.

An unexpected and distinctive peculiarity of MFLα concerns its greater accumulation in adult females. Although this sex-biased expression suggests that the subform is required during oogenesis and in the early stages of Drosophila development, it would be premature to exclude the possibility that its expression may be identical in both sexes at different developmental stages, or that a sharply restricted or a transient expression profile may have hindered its detection in adult males. Indeed, the data reported in this paper show that higher levels of expression in females are a general feature of *mfl *transcripts, either coding or non-coding, the only exception being the 1.8 kb constitutive mRNA, which has the same abundance in both sexes [7]. This observation adds further strength to a recent report that included *mfl/Nop60b *among the list of Drosophila genes that are significantly more strongly expressed in purified female germline stem cells [42]. It is also worth noting that the levels of all *mfl *transcripts peak at the major sites of cell growth and division during the Drosophila life cycle – late embryos, larvae and adult ovaries – making it plausible that gene expression might be linked to the rate of cell growth and proliferation. This hypothesis is compatible with the essential role in ribosome biogenesis [7] and with recent microarray analyses that indicated *mfl/Nop60b *to be a major target of *d-myc *induction [43]. In this light, the different levels of MFLα expression between male and female adult flies may only be coincidental, simply reflecting the different rates of protein synthesis in adult flies of different sexes. Although further experiments are required to elucidate the molecular mechanisms that regulate *mfl *expression, the intriguing pattern displayed by *snm60 *molecules (the production of which appears to be coupled to that of the 2.2 and 1.0 kb mRNAs) in proliferating intestine cells is also in good agreement with this view.

The complex molecular organization described here for *mfl *may be of general relevance, possibly unravelling aspects common to other orthologues. Although no variant *DKC1 *transcript has been characterized thus far in mammals, it is intriguing that deletion and splice site mutations in the last exon of the gene have been found in DC patients [44,45], and that a small deletion covering only the last *DKC1 *exon proved to be lethal in mouse knock-out experiments [12]. Together, these observations indicate that the 3' portion of the gene is also highly biologically relevant in mammals. Moreover, *DKC1 *-deleted alleles triggered early lethality only when they occurred on the maternal allele, revealing the existence of a maternal effect in the transmission of DC disease [12]. It is tempting to speculate that, similarly to *mfl*, mammalian *DKC1 *genes may encode still-undetected transcripts, the expression of which may account for this sex-specific effect.

Our data also show that  *mfl *introns harbour a variegated cluster of small ncRNA genes comprising an H/ACA snoRNA gene, four copies of the C/D snoRNA *DmSnR60 *and two copies of the small ncRNA of unknown function. The structure of this dense cluster conforms to the one ncRNA gene per intron rule widely observed in animal genomes, and strongly implies that several duplication events have occurred during its evolution. This coding/non-coding genetic arrangement has obvious regulatory potential: to coordinate the expression of nested ncRNAs with the protein products of the gene. Common functional roles are often shared by host genes and intron-encoded ncRNAs. In the case of *mfl*, the snoRNA *H1 *and *DmSnR60 *isoforms share obvious ribosome-related functions with their protein-coding host gene; they modify highly conserved residues on the 18S and 28S rRNAs, respectively. However, it is presently unclear whether the *snm60 *molecules represent functional entities, so their functional correlation with *mfl*, if any, remains to be proved. Nevertheless, the fine regulation of *snm60 *argues against the idea that they merely represent transcriptional noise. Even though these ncRNAs may be involved in the same pathway as *mfl*, the possibility that they may have totally unrelated functions cannot presently be excluded. In this case, it is conceivable that *mfl *may have been chosen as host merely to meet the need for a transcription rate high enough to produce a sufficient level of these molecules. Systematic mutagenic approaches are currently underway to address the biological role of these ncRNAs, and to determine the degree, if any, to which they contribute to the *mfl *phenotype. In any case, it is interesting to note that mammalian *DKC1 *genes have also been shown to host two intron-encoded H/ACA snoRNAs [34], indicating that the coding/non-coding genetic architecture may represent an additional conserved feature shared by these highly related orthologues.

## Conclusion

We report here that the Drosophila gene encoding pseudouridine synthase has a complex coding/non-coding structure. We have provided evidence that the use of different 3' end sites enables this gene not only to produce different mRNAs but also to release distinct sets of small intron-encoded ncRNAs, suggesting a potential novel role for overlapping 3'UTRs. Our data also reveal that a minor variant mRNA able to encode a distinct protein subform can be produced by this gene, suggesting that alternative splicing may have a role in regulating the expression of eukaryotic pseudouridine synthases.

## Methods

### DNA analysis and cloning techniques

Basic cloning techniques, PCR amplification, DNA and RNA extraction, manipulation and labeling, screening and sequencing techniques were carried out according to Sambrook and Russell [46]. All PCR-amplified fragments and 5' and 3' RACE products were cloned by using the pMOSBlue blunt ended cloning kit (Amersham Biosciences) and automatically sequenced (Primm).

### RNA analysis

After disruption and homogenization, total RNA was extracted from flies at various developmental stages, manually dissected ovaries or cultured S2 cells, using TRIzol Reagent according to the manufacturer's protocol (Invitrogen, Carlsbad, CA). Concentration, purity, and quality of the RNA were determined using the Hitachi U-1500 spectrophotometer at 260 nm and 280 nm and by gel electrophoresis. Polyadenylated RNA was selected by using oligo-dT polystyrene beads (GenElute™ mRNA Miniprep Kit, Sigma-Aldrich). For Northern blot analysis, 5 μg of poly(A)+ RNA or 10 μg of total RNA were electrophoresed and transferred to Hybond-NX filters (Amersham Biosciences) for hybridization. DNA probes were ^32^P-labeled using the Nick Translation Kit (Roche). Probes 1, 2 and 3 utilised in Northern blot analyses corresponded, respectively, to a 0.1 kb PCR-amplified genomic fragment generated with the oligonucleotides 5'-CACAAGAAACTTAAGGTGTG-3' and 5'- GATGTCTTGGGCAGTGTTGTACC-3' (probe 1), a 0.2 kb PCR-amplified genomic fragment generated with the oligonucleotides 5'-GAGGTAAATATTTAATAACTAAAAG- 3' and 5'-GATTCCTGTGGCATTCAATG-3' (probe 2), and a 0.6 kb PCR-amplified genomic fragment generated with the oligonucleotides 5'-CAAGCCTCAATCTTTTCGATTGCCTTTC-3' and 5'-GATGTCTTGGGCAGTGTTGTACC-3' (probe 3). The amount of RNA loaded in each lane was checked by hybridization with a probe derived from the gene coding for the *αTub84B *gene.

For 3'-RACE experiments, a first reverse transcription step was performed using an oligo dT with adapter sequence at its 5'-end (5'-GACTCGAGTCGACATCGA(T)_17_-3'). Amplification was then performed using a set of sense primers derived from the sequences of exon 5 (5'-GACCATGGTGTGGTGG-3'), exon 6 (5'-CTCTATCGCTAGTTTCTTAGGTCTTAGC-3'), exon 8 (5'-CTTCGAATAAACATAGGAATTAAGGTAAG-3') and exon 9 (5'-GGTCATGCAATATATGGACTATAAC-3'), all in association with the adapter primer (5'-GACTCGAGTCGACATCG -3') used in first reverse-transcription step. In 5' -RACE experiments, 300 ng of female poly(A)+ RNA were reverse transcribed using a splicing-specific primer spanning exon 3–9 junction (5'-CCTAATCAACAAATCCATATTTCGGG-3') to amplify the 5' region of the alternatively spliced transcript. An A-tailing step was carried out to attach an oligo-dA tail to the 3' end of the cDNA with terminal transferase (Roche Molecular Biochemicals) and specific cDNAs were then amplified by two rounds of PCR. The first round was performed with a gene-specific primer from exon 3 (5'-GGGCACCACGCAGCTTCTC-3') and the same oligodT-adaptor primer used in 3' RACE experiments, while in the second reaction we used a nested primer from exon 3 (5'-GGGACTTCACCAGACGGG-3') and the same adaptor primer previously described for 3' RACE. The amplification products obtained (500 bp) were cloned into the pMOSblue vector and sequenced. To clone MFLα cDNA, the partially overlapping PCR products of the 3' and 5' RACE experiments described above were digested at the common *Bam*H1 site present at exon 2 and fused in a ligase reaction (T4 DNA-ligase USB) to compose a virtual full length cDNA sequence. The reconstituted cDNA sequence was cloned into the pMOSblue vector and sequenced to check absence of internal stop codons.

For RT-PCR, we utilised poly(A)+ RNA preparations from male and female adult flies and various combinations of upstream primers derived from exon 2 (primer P1: 5'- CTTCCAAATCAAGCCCTCCTCCAAG-3'), exon 5 (5'-GCCAGCAGCTCAAGAAGTCTCCCC-3') and exon 6 (5'-GTGTAGAAGTGCATACAAATTAC-3') with downstream primers derived from exon 10 (5'-GATTCCTGTGGCATTCAATG-3'), exon 11 (5'-GCAATAAAGTGTCATGCG-3') and exon 12 (primer P2: 5'- GATGTCTTGGGCAGTGTTGTACC-3').

To confirm presence of the alternatively spliced mRNA, in order to avoid amplification from genomic DNA contamination we utilised a common 5' primer from exon 2 (primer P1) in combination with two 3' primers spanning splicing specific exon-exon junction (primer P3, spanning exon 3–9 junction: 5'-CCTAATCAACAAATCCATATTTCGGG-3'; primer P4, spanning exon 5–6 junction: 5'-GGTGCTTAACTTGCGTTTGCTGGG-3'). As control of the RNA quantity we also reverse-amplified in each sample the *αTub84B *gene transcript, using a forward primer derived from exon 1 (5'-GTGAAACACTTCCAATAAAAACTCAATATG-3') in combination with a reverse primer derived from exon 2 (5'-CCAGCAGGCGTTTCCAAT-3'). The amplified DNA products were analysed on 1.5% agarose gel, eluted and purified before cloning and sequencing.

The 5' end of *DmSnR60 *isoforms *a *and *c *was determined by primer extension experiments, using 50 μg of total RNA and a primer complementary to nucleotides 181–209 (5'-TTGCGTTATTTTCAGTAAGACCAATCTCG-3') of intron 6, or to nucleotides 93–117 (5'-GAAATTCTTTTCAGTAAGACCAATC-3') of intron 8. The 5' end of *snm60 *isoforms *a-b *were similarly determined, using a primer complementary to nucleotides 193–224 (5'-GGCATCGTTGTTATTGTATTGGCCTAAATGC-3') of intron 10 or to nucleotides 150–180 (5'-CACTTAGAACCACATCTGGTTAAAACACAC-3') of intron 11.

### GenBank accession numbers

The nucleotide sequence of the 2.2 kb and the 1.0 kb mRNAs have been submitted to the GenBank database under accession numbers DQ857345 and DQ 857346, respectively. GenBank accession numbers of the nucleotide sequences of *snm60 a *and *b *isoforms are DQ142641 and DQ142642; sequences of *DmSnR60 *isoforms (*a-d*) are found in the GenBank database under the accession number AY805216. *SnoH1 *sequence is found in GenBank at the AF089836 number, while *mfl *genomic sequence is found in GenBank at AF097634. MFL amino acid sequence is available at the GenBank accession number AAD19897.

### Quantitative Real Time RT-PCR (qPCR)

Poly(A)+ RNA (800 ng) was reverse transcribed using 250 ng random hexamers, 100 U SSII reverse transcriptase, 10 mM DTT, and 1X First-Strand Buffer (Invitrogen, Carlsbad, CA) at 42°C for one hour in a volume of 20 μL (Mycycler, BioRad, 8 Hercules, CA). Quantitative analysis was performed by using the iQ™ 5 Multicolor Real-Time PCR Detection System (Bio-Rad). The PCR reactions were performed in a final volume of 15 microliters using 1 microliter of cDNA, 5 pmol of each primer and 7.5 microliter of iQ™ SYBR Green Supermix 2X (Bio-Rad). PCR cycling profile consisted of a cycle at 95°C for 3 min and 40 two-step cycles at 95°C for 10 s and at 60° C for 30 s. Quantitative real time PCR analysis was carried out using the 2(-Delta Delta C(T)) method (2^-ΔΔCt^) [47]. Primers were chosen using Primer Express 2.0 software (Applied Biosystems, Foster City, CA) for optimum use in qPCR. A BLASTN search was performed against GenBank to ensure that all primers were unique to the gene of interest. To avoid amplification from genomic DNA contamination, all primer sets derived from different gene exons or spanned a specific exon-exon junction (see below). In all qPCR experiments the data were normalized to the expression of the Drosophila *αTub84B *housekeeping gene. Negative controls included omission of template dissociation curves, gel analysis and sequencing of certain PCR products confirmed gene specific product amplification. PCR oligo-primers were: *mfl *Ex 3 forward primer: 5'-GTTGCGCGTTCGTACTGTCTAC-3'; *mfl *Ex 4 reverse primer 5'-CCTCGCAACTAACCCAAAAAAC-3'; *mfl *Ex 3/9 junction reverse primer 5'-TTGCATAACCTAATCAACAAATCCA-3'; α-*Tub*84B Ex 1/2 forward primer 5'-GTGAAACACTTCCAATAAAAACTCAATATG-3'; α*-Tub84*B Ex 2 reverse primer 5'-CCAGCAGGCGTTTCCAAT -3'. Three different RNA preparations were tested for each sample, and each reaction was run in triplicate. Data are representative of three independent experiments.

### Detection of ribose-methylated nucleotides

rRNA 2'-O-ribose methylation was determined by reverse transcription at low dNTP concentration essentially as described by Maden et al. [35].

### Antibodies and immunochemistry

Two peptides, peptide 1 (N2H-CADVEVRKEKKKKKI K-CONH2) and peptide 2 (N2H-CHG SSPLNRDIKEYH K- CONH2), were synthesized. These peptides were selected from tracts of the N-terminal region common to MFL and MFLα proteins that are not highly conserved between species. Both peptides contained an extra cysteine on N-terminus to aid in affinity purification and were conjugated (to keyhole limpet hemacyanin, mixed together). Polyclonal antibodies against these two peptides were produced by injection of rabbits (Eurogentec, EGT group) and used in Western blot analyses after 1:500 dilution in TBS tween. Chemiluminescent detection was performed using HRP conjugated anti-mouse antibodies (Sigma-Aldrich) diluted 1:5000 and subsequently detected by a film exposure. The ECL-Advance™ Western Blotting Detection Reagents (Amersham Biosciences) were used. The protein ladder used in our experiments was the Precision Plus Prestained Protein Standard Dual Colour (Biorad Laboratories).

### In situ hybridization

Whole mount in situ hybridization was performed using single-stranded DIG-labeled probes obtained by PCR, as described previously [7]. Preparations were observed through a microscope model Eclipse E1000, Nikon, with plan fluor Phl20x/0.50 objective lenses. The microscope was equipped with a digital camera (Nikon model DXM 1200 F) and with Nikon ACT1 acquisition software.

## Authors' contributions

SR designed and performed most of the experiments; she carried out the Northern and bioinformatic analyses, 5' and 3' RACE, RT-PCR, 2'-O-methylation mapping and *in situ *hybridization experiments, took care of handling the Drosophila, and helped to organize the data and draft the manuscript. GT prepared the cell extracts and performed the western analyses. EG helped to map the 2'-O-methylations on rRNA, participated in the bioinformatic analyses and gave useful suggestions. MT carried out the S2 cell culture and RT quantitative PCR experiments and contributed to the western analysis. MF conceived and coordinated the study, planned the experiments, analysed the data and wrote the manuscript. All authors read and approved the final version of the manuscript.
